# Prevalence of medical students’ burnout and its associated demographics and lifestyle factors in Hong Kong

**DOI:** 10.1371/journal.pone.0235154

**Published:** 2020-07-10

**Authors:** K. P. Lee, Nelson Yeung, Carmen Wong, Ben Yip, Lawrence H. F. Luk, Samuel Wong

**Affiliations:** JC School of Public Health and Primary Care, The Chinese University of Hong Kong, Hong Kong, Hong Kong; Universitat de Valencia, SPAIN

## Abstract

**Background:**

Burnout causes personal suffering and adverse professional consequences. It is prevalent among medical students, although the relationship between burnout and lifestyle factors are understudied in Chinese medical students. Thus, this study aims to (i) estimate the prevalence of burnout among medical students in Hong Kong (HK) and (ii) delineate the relationship between burnout and various lifestyle factors.

**Method:**

1,341 students were invited to complete a questionnaire from September to December 2017. Burnout was measured by the Maslach Burnout Inventory. Lifestyle factors including drinking habit, sleep habit and quality, and exercise level were assessed by validated instruments, including Alcohol Use Disorder Identification Test (AUDIT-C), Pittsburgh Sleep Quality Index (PSQI), and Godin-Shephard Leisure-Time Physical Activity (GSLTPA), respectively. Smoking status and use of self-medications were also inquired into, while demographic data was self-reported. Prevalence of burnout with confidence intervals was calculated. Difference in lifestyle and demographic data in students with or without burnout, were compared by t-test and Chi-square/Fisher’s exact test. From this, all associations with significant p-value at p<0.1 were entered into the multiple logistic regression model.

**Results:**

731 students (55.6%) responded to the questionnaire. Prevalence of burnout was 27.9% (95%CI: 24.6%-31.5%). Only 3 students in the whole sample smoked; and 6.6% of students drank weekly but rarely drank more than 2 drinks per week. 6.3% and 2.3% self-medicated themselves with medications to improve their sleep and concentration, respectively. Using a multiple logistic regression model, only sleep quality and exercise level were significantly associated with the presence of burnout.

**Conclusion:**

Around a quarter of medical students in HK suffered from burnout. Burnout was found to be significantly associated with sleep quality and physical exercise. The study also highlighted that HK medical students lived very different lifestyles from those from other countries. More research is needed to design and establish the effectiveness of lifestyle interventional programs that enhance exercise level and sleep quality.

## Introduction

Burnout is a state of emotional and physical exhaustion and is common among medical students [1]. Besides causing personal suffering, medical student burnout is associated with suicidal ideation even after controlling for the presence of depression [1,2], thoughts of dropping out of medical school [1,3], decreases in empathy [4], poorer academic results [3], alcohol dependence/abuse [5], and professional misconduct [6]. For example, students with high burnout are more likely to report normal physical findings when the relevant physical exam is omitted [6]. Research suggests that burnout begins in medical school and persists after graduation [4,7], and burnout in doctors is associated with medical errors [8–11], poorer patient outcomes [12] and rapid turnover of doctors [13,14]. The prevalence of suicidal ideation among medical students worldwide is alarmingly high at 11.1% [15] and burnout students are three times more likely to have suicidal ideation [2]. A recent systematic review found that around 25% to more than 70% of medical students suffered from burnout, which was associated with poorer academic performance and lack of social support [16].

Many different aspects of medical curriculums promote burnout. At a personal level, teachers and students believe that the curriculum must be extremely rigorous, requiring sacrifice of personal time and resources [17]. At a training level, medical students need to repeatedly face stressful trainings that are unique, including cadaver contact and witnessing death [4]. Similarly, teachers and residents can sometimes act uncharitably towards medical students [18]. At an institutional level, evaluation of curriculum often does not include evaluation of its’ mental effects on students [17]. Furthermore, medical students are more socially exclusive and isolated from other students [4].

While many factors, such as life events and social support are found to be associated with burnout, lifestyles may be the most modifiable factor [16]. However, only a few studies investigated the relationship between various lifestyle factors and burnout in medical students; and the latest systematic reviews also did not describe this relationship [1,16]. There are only two studies that directly described multiple lifestyles factors and its’ relationship with burnout in medical students in the same study. Cecil *et al*. surveyed 356 medical students in the United Kingdom and found that various lifestyle factors are associated with a subscale of burnout, but they did not report the lifestyle factors that were associated with burnout [19]–for example, physical activity and smoking was only associated with high emotional exhaustion scores and binge drinking was only associated with low personal accomplishment scores. Similarly, a relatively small study involving 224 postgraduate medical students in China found that burnout was associated with smoking, drinking and other demographic data [16]. Although numerous studies reported that burnout was associated with one or two particular lifestyle factors, different lifestyle factors are interrelated and these studies could not indicate the most important lifestyle factor. For example, a few studies only reported relationship between burnout and alcohol [5,20]; similarly, a few other studies only described the relationship between burnout and sleep [21].

Previous studies in the Southeast Asian region (including Hong Kong, Singapore and Malaysia) focused on the presence of psychiatric illness or general emotional well-being in medical students but burnout amongst medical students remains understudied [22–24]. In addition, previous research shows that university students in HK have different lifestyles when compared to those of overseas students; for example, they are less likely to use alcohol than their counterparts in European countries [25]. Therefore, results from other countries may not be applicable to students in China and Hong Kong. However, the prevalence of burnout, and its’ relation to lifestyles and relationships has not yet been studied in medical students in Hong Kong.

As suggested by The Association of American Medical Colleges, medical education should include programs to aid ‘the health and wellbeing of learners’ [26]: the two medical schools in HK are developing wellness programs for their medical students to relieve or prevent burnout. Data about medical students’ burnout can help HK medical schools to develop tailored interventions, to enhance awareness of burnout among teachers and students, and to provide baseline data for periodic monitoring of changes in burnout after various interventions in the future.

The Chinese University of Hong Kong (CUHK) is one of two Universities in HK that provides medical training, including 3 years of pre-clinical studies (in the first 3 years) and 3 years of clinical practice training. At the time when this study was conducted, there was no well-structured wellness program to reduce students’ burnout in the medical school.

### Objectives and hypothesis

This study aimed to delineate the prevalence of burnout in medical students in CUHK and examine demographic and lifestyle factors associated with burnout. We hypothesized that the prevalence of burnout in HK medical students was similar to medical students’ burnout prevalence worldwide (~50%) [1], and that burnout was associated with various lifestyle factors. Lifestyle factors including sleep, exercise, smoking and alcohol use were assessed, because these factors were shown to be related to burnout in previous studies [19]. As it has been reported that university students in other countries commonly self-medicated psychiatric medications, including sleep medications and stimulants [27,28] to improve academic performance, these data were also collected to describe the situation in Hong Kong.

## Methods

This research employed a cross-sectional survey, which invited all medical students in CUHK (n = 1341) to complete a questionnaire during the first semester between September to December 2017, so that results would be least affected by examinations. Burnout was measured by the validated Maslach Burnout Inventory [1,29]. Lifestyle factors were assessed by the validated instruments Alcohol Use Disorder Identification Test (AUDIT-C) for drinking habit; Pittsburgh Sleep Quality Index (PSQI) for sleep habit and quality; and Godin-Shephard Leisure-Time Physical Activity (GSLTPA) Questionnaire for exercise level and smoking status, by asking if they ever smoked, and the duration and the packs per day if they did smoke. Self-medication (including sleeping medications and medications to enhance concentration) use was also asked. In addition, demographics of the participants such as age, sex, year of study in medical school, marital status, and whether they lived with their family were asked. While most students are admitted to the medical school via the Joint University Programmes Admissions System (JUPAS) by sitting a joint examination organized by the Government, students from other countries and international secondary schools can enter the medical school by non-JUPAS means by taking other international examinations. There were no identifying details on the questionnaire, and participation was entirely voluntary. This study was approved by the Chinese University of Hong Kong Survey and Behavioural Research Ethics Committee (reference no.: 108–17).

### Specific instruments

#### MBI

The Maslach Burnout Inventory (MBI) is a 22-item questionnaire and is considered the gold standard for assessing burnout, as revealed by a systematic review [1,29]. Each question can be rated using a 7-point Likert scale ranging from ‘never’ to ‘everyday’. It consists of 3 subscales which are scored individually: personal accomplishment (PA), emotional exhaustion (EE) and depersonalization (DP) subscale. Burnout is defined as low in PA but high in EE and DP. Cutoffs of each subscale were established and used (See S1 Appendix) [30].

#### AUDIT-C

AUDIT-C is used to assess drinking habit in college students [31]. The questionnaire consists of 3 questions, with each question scored from 0–4 (for question 2, both answers ‘0 drinks’ or ‘1–2 drinks’ score 0), making the possible total score range from 0 to 12. The last question ‘how often do you have 6 or more standard drinks on one occasion’ (the possible answer ranged from ‘never’ to ‘almost daily or daily’) can be analyzed individually for presence of binge drinking [32].

#### GSLTPAQ

GSLTPAQ is a 4-item questionnaire that was validated in healthy adults [33] and is employed to assess exercise habit among college students [34], and is used in HK [35]. The time spent exercising at different intensities weekly was asked, and a total score was calculated using the following formula: Weekly leisure activity score = (9 × Strenuous) + (5 × Moderate) + (3 × Light). It also includes an extra question about self-rated exercise frequency during a typical week–possible answers included ‘often’, ‘sometimes’ and ‘rarely or never’.

#### PSAQI

It was developed in the USA [36] with good sensitivity and specificity, and has since been validated and found reliable in Chinese settings [37]. The total score can be calculated according to specific a protocol, and a total score of more than 5 signifies poor sleep quality [38]. The last question seeks to probe self-rated sleep quality: ‘During the past month, how would you rate your sleep quality overall?’ and can give a global self-reported sleep quality in addition to the total PSQI score.

#### Statistical analysis

Prevalence of burnout with confidence interval was calculated. Descriptions for demographic data and lifestyle behavior data were computed for mean with standard deviation and percentages. Differences in lifestyle and demographic data in students with or without burnout, were compared by t-test for continuous data and Chi-square/Fishers’ exact test for categorical data. All the associations with significant p-value at p<0.1 were entered into the multiple logistic regression to describe the relationship between burnout and lifestyle/demographic data.

For secondary analysis, same statistical tests and logistic regression method were used to describe the relationship between each subscale (PA, EE, DP) in the MBI and lifestyle and demographic data. These relationships were commonly reported in other international studies [19].

## Results

### Participants’ demographics (Table 1)

**Table 1:** Demographics of participants.

Characteristics	Mean	Percentage
Age (years)	20.54 (SD = 2.07)	
Gender (male/female)		
Male		44.2%
Female		55.8%
Year of Study		
Year 1		16.2%
Year 2		39.5%
Year 3		12.3%
Year 4		20.3%
Year 5		22.8%
Year 6		5.1%
First degree before entering medical school		
Yes		92.1%
Place of living		
In campus		34.3%
Hospital (dormitory)		12.0%
Home		53.0
Others		0.7%
On loan		
Government loan		11.7%
Other loan		0.7%
No		87.7%
Any scholarship		44.8%
Marital Status		
Single		98.2%
Married		0.4%
Divorced		0.1%
Others		1.2%
Entry via JUPAS?^		
Yes		58.8%
Previously studied abroad?		
Yes		17.6%
In GPS program?^^		
Yes		12.4%
High burnout?		
Yes		27.9%
Emotional Exhaustion level		
High		49.3%
Medium		29.2%
Low		21.6%
Depersonalization level		
High		53.8%
Medium		22.8%
Low		23.4%
Personal accomplishment level		
High		5.1%
Medium		23.2%
Low		71.1%
Smoking?		
Yes		0.4%
Self-prescribe sleep medication		
Yes		6.3%
Self-prescribe concentration medication		
Yes		2.3%
Alcohol use frequency		
Never		29.7%
Monthly or less		45.5%
2–4 times/month		18.3%
2–3 times/week		5.2%
4–5 times/week		1.4%
Binge drinking (≥6 drinks) in last 1 year		
Never		73.1%
Less than monthly		21.0%
Monthly		4.9%
Weekly		0.8%
Daily or almost daily		0.1%
Regular exercise in a typical week		
Often		19.9%
Sometimes		52.9%
Never/rarely		27.2%
Poor sleep on PSQI cut-off		
Poor sleep		31.5%
Self-reported sleep quality		
Very good		20.7%
Fairly good		61.4%
Fairly bad		16.9
Very bad		1.0%
Response rate		
Year 1		63.0%
Year 2		69.5%
Year 3		41.4%
Year 4		65.9%
Year 5		79.4%
Year 6		16.2%
Total		55.6%

^ Most students in Hong Kong enter University programs by sitting a unified examination called Diploma of Secondary Education (DSE) and were admitted to Universities by a central system called Joint University Program Admission System (JUPAS). But significant proportion of students admitted to medical schools receive secondary education in international schools and other countries, and they can enter the medical school by non-JUPAS means.

^^ CUHK is unique in that it offers the choice of Global Physician Scheme (GPS) to its’ students, in which the academically promising students receive extra training for research, for leadership development and for international exposure.

A total of 1,341 questionnaires were distributed and 731 students responded, which constituted a response rate of 55.6%. The mean age of respondents was 20.5 years old (S.D. = 2.07). The majority were female (55.8%), studying their medical degree as their first degree (92.1%), living at home rather than on campus (53%), were not taking out a loan (87.7%), had not received scholarship (44.8%), and were not married (98.2%).

### Burnout prevalence (Table 1)

Prevalence of burnout in our participants was 27.9% (95%CI: 24.6%-31.5%). The proportions of students who scored high in EE, DP and low in PA were 49.3% (95%CI: 45.5%-53%), 53.8% (95%CI: 49.9% -57.5%), and 71.1% (95%CI: 67.5%-74.4%), respectively.

### Lifestyle of participants (Table 1)

The vast majority of our participants did not smoke (99.6%). Among the 3 students (out of more than 700 respondents) who smoked, the smoking pack years ranged from 0.1 to 0.7 only. As for alcohol use, only a minority (6.6%) drank weekly; and most participants did not drink more than 2 drinks when they did drink. However, 26.9% reported binge drinking (as defined by more than 6 drinks on any occasion) during the last year.

6.3% (n = 46) and 2.3% (n = 17) of participants self-medicated with sleep medications and medications to enhance concentration or to their ability to study in the last 12 months. A quarter of participants did not exercise regularly (25.3%), while around a third (31.5%) of participants had poor sleep according to PSQI scores cut off at 5.

### Relationship between lifestyle and burnout (Tables 2 and 3)

**Table 2:** Relationship between demographics/lifestyle factors (continuous data) and burnout.

Variables	With burnout	Mean	p-value
Age	Yes	20.7	0.590
	No	20.6	
GSLTPAQ score	Yes	28.1	0.034
	No	32.8	
PSQI score	Yes	6.359	<0.001*
	No	4.35	
AUDIT score	Yes	2.51	0.073
	No	2.38	

**Table 3:** Relationship between demographics/lifestyle factors (categorical data) and burnout.

Variables		Proportion with burnout	p-value
Gender			
	Male	29.5%	0.406
	Female	26.6%	
Clinical years?			
	Pre-clinical years	25.2%	0.116
	Clinical years	30.7%	
Years of study			
	Year 1	30.0%	0.197
	Year 2	22.8%	
	Year 3	23.0%	
	Year 4	27.4%	
	Year 5	31.1%	
	Year 6	42.4%	
Medical degree as first degree?			
	Yes	29.1%	0.03*
	No	15.1%	
Place of living			
	Campus	25.7%	0.02*
	Hospital (dormitory)	39.8%	
	Home	26.1%	
	Others	100%	
On loan?			
Government loan, means		37.9%	0.513
Government loan, non-means		32.7%	
	Other loans	33.3%	
	No	27%	
Received scholarships?			
	yes	27.6%	0.858
	no	28.2%	
Marital status			
	single	28.1%	0.221
	Married	0%	
	divorced	100%	
	others	14.3%	
Enter by JUPAS?^			
	Yes	31.7%	0.008*
	No	22.2%	
Once studied aboard?			
	Yes	24.6%	0.359
	No	28.8%	
In GPS program?^^			
	Yes	30.1%	0.656
	No	27.7%	
Self-prescribed sleep medication?			
	Yes	39%	0.113
	No	27.5%	
Self-prescribed concentration medication?			
	Yes	35.3%	0.513
	No	28.1%	
Physical exercise in a typical week?			
	Often	13.7%	<0.001*
	Sometimes	28.6%	
	Rarely/never	37.6%	
Self-rated sleep quality			
	Very good	13.2%	<0.001*
	Fairly good	27%	
	Fairly bad	45.3%	
	Very bad	83.3%	
Smoking?			
	Yes	33.3%	0.628#
	No	28%	
Binge drinking in last year?			
	Never	27.2%	0.842
	<monthly	29.7%	
	Monthly	34.4%	
	Weekly	25%	
	Daily/almost daily	0%	
Used alcohol at least 4-5/week			
	Yes	62.5%	0.029*
	No	27.5%	

*Denotes statistically significant relationship.

#Fish exact test used because n<5 in some cells.

^ Most students in Hong Kong enter University programs by sitting a unified examination called Diploma of Secondary Education (DSE) and were admitted to Universities by a central system called Joint University Program Admission System (JUPAS). But a significant proportion of students admitted to medical schools receive secondary education in International schools and other countries, and they can enter the medical school by non-JUPAS means.

^^ CUHK is unique in that it offers the choice of Global Physician Scheme (GPS) to its students, in which the academically promising students receive extra training for research, for leadership development and for international exposure.

Burnout was not associated with age, gender, years of study, whether they carried a debt/loan, received any scholarship, their marital status, whether they once studied aboard, if they joined the GPS program, the use of self-medications (for sleep/concentration), binge alcohol drinking, and smoking status.

Participants with burnout had lower exercise levels as shown by GSLTPAQ scores (mean: 32.8 vs 28.1; p = 0.034); participants who reported they often did exercise were less likely to experience burnout than those who reported they ‘sometimes’ or ‘rarely’ exercised (13.7% vs 31.7%; p<0.001); similarly, participants who reported performing regular exercise were less likely to experience burnout than those who reported they ‘rarely’ exercised (24.6% vs 37.6%; p = 0.001). Furthermore, participants who were suffering from burnout were more likely to use alcohol more than 4–5 times per week (62.5% vs 27.5%; p = 0.029), although the total AUDIT score did not differ between those with and without burnout. Students who experienced burnout were also more likely to have their medical degree as their first degree (29.1% vs 15.1%; p = 0.03), more likely to enter the program via JUPAS (31.7% vs 22%; p = 0.08) and more likely to live in the hospital dormitory (p = 0.011). High burnout level was also highly associated with self-reported sleep quality (p<0.001) and students with burnout had significantly higher PSQI scores (mean: 4.35 vs 6.36; p<0.001).

### Logistic regression model to identify the most predictive lifestyle factors to burnout (Table 4)

**Table 4:** Logistic regression model for medical students’ burnout and their demographics/lifestyle factors.

	OR	95% C.I.		
		Lower	Upper	p-value
MBChB as first degree	2.258	0.900	5.666	0.083
How often did you have a drink containing alcohol in the past year?				
Never	ref			
Monthly or less	1.022	0.648	1.614	0.925
2–4 times a month	0.960	0.534	1.726	0.892
2–5 times/week	1.088	0.485	2.440	0.837
Living in hospital versus others	1.684	0.957	2.963	0.071
Entry through JUPAS	1.341	0.871	2.066	0.183
Receives scholarship	0.824	0.556	1.221	0.334
GSLTPAQ score	1.003	0.994	1.011	0.560
During a typical 7-Day period (a week), in your leisure time, how often do you engage in any regular activity long enough to work up a sweat (heart beats rapidly)?				
Often	ref			
Sometimes	2.621	1.381	4.974	0.003*
Never / rarely	4.047	1.948	8.404	<0.001*
During the past month, how would you rate your sleep quality overall?				
Very good	ref			
Fairly good	2.079	1.171	3.691	0.012*
Fairly bad / Very bad	6.049	3.090	11.840	<0.001*

*Represent statistically significant relationship.

Because both PSQI score and self-reported sleep quality were significantly associated with burnout and these two domains were highly correlated, self-reported sleep quality was entered into the logistic regression model because this domain had less missing data. The logistic regression using PSQI instead of self-report sleep quality is included in the S1 Appendix, which showed similar results.

When all demographic and lifestyle factors with a significant association to burnout (as defined as p<0.1) were entered into a logistic regression model, only the self-reported sleep quality (fairly bad to very bad quality vs very good quality: OR 6.049; p<0.001), and self/-reported exercise level (never exercise vs often exercise: OR 4.047; p<0.01) were still significantly associated with burnout level. The overall fitness of the model is now accessed by Hosmer-Lemeshow test for calibration (p-value = 0.503) and area under curve statistics (C-statistics = 0.696, 95%CI: 0.651–0.741) for discrimination.

Secondary analysis was conducted similarly for emotional exhaustion(EE), depersonalization(DP) and personal accomplishment(PA) subscale in the MBI. The logistic regression analysis revealed that low PA was predicted by poorer self-rated sleep quality (very bad/fairly bad versus very good; OR = 1.988, p = 0.034), being male (p = 0.04), receiving a scholarship (OR 1.522; p = 0.033) and being admitted to medical school by non-JUPAS (OR 0.607 for JUPAS students, p = 0.029); high EE was predicted by sleep quality (very bad/fairly bad versus very good; OR 0.232, p<0.001) and low self-reported exercise frequency (never/rarely versus often; OR 0.346, p <0.001); and high DP was predicted by self-reported sleep quality (fairly bad/very bad versus very good; OR 0.38, p<0.001) (see S1 Appendix).

## Discussion

This first study of burnout of medical students in Hong Kong found that more than a quarter of students suffered from burnout, and sleep and exercise were the most important lifestyle factors associated with burnout. Furthermore, demographic data (e.g., age, sex, years of study) were not important determinants of burnout. The prevalence of burnout in our study was similar or less than that reported in other studies [2,16,19]. However, different studies used different instruments or definitions to define burnout, which made direct comparison difficult [2,16]. For example, Dyrbye *et al*. defined burnout as having high scores in EE or DP and they found that half of students had burnout [2]; using their criteria, the prevalence of burnout in our participants raised to 65.7% (result not shown), which is similar to that reported in international studies [1]. In this study, we used MBI scale to assess burnout, which is considered the gold standard [1]; while burnout was originally defined as high EE and DP and low PA rather than one of these components [1]. Studies that used the same criteria to define burnout appeared to report similar burnout prevalence with the current study [19].

Our study also shows that medical students in HK have substantially different lifestyles from those reported in other countries. While few of our participants were regular drinkers and they rarely drank more than the recommended drinking limits, European studies found that alcohol abuse was common among medical students. For example, Newbury-Birch *et al*. conducted a survey in the United Kingdom and found that alcohol abuse was common among medical students–around 41% of their male participants and 19% of their female participants drank above the recommended limits during a typical week [39]. Similarly, an Irish study found that more than 70% of medical students regarded themselves as current alcohol user and more than 50% of these users might be considered to be addicted to alcohol [40]. The prevalence of smokers in our sample was also substantially lower than that found in medical students in most parts of the world, including China [41–44]. The prevalence of use of self-prescribed medications for sleep (6.3%) and concentration (2.3%) were comparable to their prevalence in university students in Middle Eastern countries [27], but were lower than that reported by medical students from the USA [28]. These data confirm that medical students in HK have different lifestyles patterns and that results from international studies on burnout may not be applicable to these medical students.

This is one of the first studies that investigated the relationship between burnout and numerous lifestyle factors in the same study and, to our knowledge, the first one to include self-medications as one of the lifestyle factors. Cecil *et al*. had reported the relationship between lifestyle factors and subscales of burnout, and found that physical activity and smoking was only associated with emotional exhaustion and binge drinking was only associated with personal accomplishment scores [19]. Smoking, however, was not associated with burnout in our study, likely due to fewer smokers in our sample [19]. On the contrary, sleeping habits were associated with all 3 subscales of burnout in the current studies, and exercise and sleeping habits are associated with burnout. While the relationship between burnout and lifestyle was not studied in medical students in China, physical exercise were associated with better quality of life in medical students there [45].

Another finding was that burnout was not associated with any of the demographic data in the multiple logistic model, although burnout was more prevalent in students who were studying for their first degree or who were living in a dormitory located in the hospital. Many of the relationships probed between burnout and demographic data were not significant, possibly due to low prevalence of several characteristics–for example, very few of our participants were married. Students who were studying for their second degree might have learned techniques to cope with stress in their first degree; and students who lived in the hospital were more likely to be in their clinical rotation and might have longer studying hours. In contrast to previous studies [1], similar levels of burnout were found in students who were in their pre-clinical and clinical years, as it was commonly reported that burnout might be more common when students were exposed to patients and their sufferings. Students from different years of study may have different sources of burnout [1]. While some studies suggested that student burnout was more prevalent in later stages in the medical curriculum [1], this relationship was not consistent across different studies [20].

These results were important because, by understanding students’ lifestyle and level of burnout, tailored interventions can be developed. In particular, our results suggest that interventions should encourage physical exercise and good sleep hygiene to reduce burnout in medical students; yet, there is a lack of relevant interventional studies [46]. Other lifestyle intervention studies have investigated the effectiveness of mindfulness-based interventions, which may also improve sleep quality to reduce students’ burnout, and found conflicting results [47,48]. More studies are needed to design structured lifestyle programs and investigate their effectiveness to reduce burnout.

Despite our findings, several limitations must be discussed. Firstly, as a limitation common to cross-sectional studies, causal relationships cannot be confirmed. For example, while physical activities were shown to improve the chances for avoiding burnout [49], it is also likely that students with less burnout will do more exercise; similarly, a bidirectional relationship was implied between burnout and sleep [50]. Longitudinal studies and qualitative studies could further clarify the direction of these associations, although only few longitudinal studies have been conducted [3]. Our group plans to conduct a qualitative study with the medical students based on these results.

Secondly, our response rate was modest at 55.6%. Questionnaires were distributed at the end of a whole-class lecture. Students would not be able to complete the questionnaire if they skipped class. While response rate was good for year 1–5 (41.4–79.4%), the response rate from year 6 students was poor (16.2%) because many students skipped the lecture. Yet, the overall response rate remained comparable or higher to those of other international studies (typically from around 22 to 55%) [18,20,21]. Ideally, random sampling of non-responders could investigate possible selection bias. However, this is impossible because the survey was conducted anonymously, which aimed to encourage accurate answers to sensitive questions (e.g., self-medications and excessive drinking). Innovative and acceptable ways to collect data from medical students needs to be sought, e.g., through the use of mobile apps.

A suboptimal response rate might have selected students with lower burnout levels and biased our prevalence estimation, i.e., those experiencing burnout were more likely to skip class and/or refuse to answer the questionnaire. Furthermore, our estimation of the prevalence of burnout could represent an underestimation, because the results were obtained at the beginning of the semester and burnout prevalence might be higher if the data were obtained near the examination period. Also, despite sharing similar curriculum structure, number of students and culture, participants were recruited solely from CUHK and the results may not be generalizable to participants in another medical school in HK. Lastly, although we have included many important existing demographic and lifestyle factors that are associated with burnout, we cannot include all possible parameters; most of the parameters were self-reported and prone to reporting bias, but this is a limitation common to all cross-sectional survey studies.

Furthermore, future studies may also include other relevant parameters such as depressive symptoms, anxiety symptoms, and mindfulness levels, which were not measured by the current study. While mood disorders (including anxiety disorders and depression) are clinical diagnoses and burnout described a state of mental exhaustion due to occupation, they shared similar symptoms and there is significant overlap between depression and burnout syndrome [51]. Therefore, it is possible that some students suffering from burnout are also suffering from depression; and the confounding effect of mood disorders on our results cannot be excluded. Similarly, the relationship between baseline mindfulness level and burnout level is not currently clear. Existing studies have also investigated other psychosocial factors but these are less modifiable, including personality, hours of work per week, social network, peer support, and recent life events [18,52]. Inclusion of all these factors may enhance the overall moderate predictive power of the current regression model, but may limit the response rate.

## Conclusion

Around a quarter of medical students in HK suffered from burnout and medical students in HK have different lifestyle patterns when compared to other countries, particularly those in European nations. In this study, burnout was found to be significantly associated with sleep quality and physical exercise. More research is needed to design and establish the effectiveness of lifestyle interventional programs that enhance exercise level and sleep quality.

## Supporting information

S1 Appendix (SAV)
